# Food and beverage manufacturing and retailing company policies and commitments to improve the healthfulness of Canadian food environments

**DOI:** 10.1186/s12889-024-19864-1

**Published:** 2024-09-05

**Authors:** Alexa Gaucher-Holm, Jasmine Chan, Gary Sacks, Caroline Vaillancourt, Laura Vergeer, Monique Potvin Kent, Dana Lee Olstad, Lana Vanderlee

**Affiliations:** 1 École de nutrition, Centre NUTRISS (Nutrition, santé et société), Institut sur la nutrition et les aliments fonctionnels, Université Laval, 2425 Rue de L’Agriculture, Québec City, QC G1V 0A6 Canada; 2 Global Centre for Preventive Health and Nutrition (GLOBE), Institute for Health Transformation (IHT), Deakin University, Geelong, VIC Australia; 3 School of Epidemiology and Public Health, University of Ottawa, Ottawa, ON Canada; 4 Department of Nutritional Sciences, University of Toronto, Toronto, ON Canada; 5 Department of Community Health Sciences, Cumming School of Medicine, University of Calgary, Calgary, AB Canada

**Keywords:** BIA-Obesity, Food and beverage industry, Grocery retail, Accountability, Food environment, Nutrition policy, Non-communicable disease

## Abstract

**Background:**

Food and beverage companies play a central role in shaping the healthfulness of food environments.

**Methods:**

The BIA-Obesity tool was used to evaluate and benchmark the specificity, comprehensiveness and transparency of the food environment-related policies and commitments of leading food and beverage manufacturing and retailing companies in Canada. Policies and commitments related to the healthfulness of food environments within 6 action areas were assessed: 1) corporate nutrition strategy; 2) product (re)formulation; 3) nutrition information and labelling; 4) product and brand promotion; 5) product accessibility; and 6) disclosure of relationships with external organizations. Data were collected from publicly available sources, and companies were invited to supplement and validate information collected by the research team. Each company was then assigned a score out of 100 for each action area, and an overall BIA-Obesity score out of 100.

**Results:**

Overall BIA-Obesity scores for manufacturers ranged from 18 to 75 out of 100 (median = 49), while scores for retailers ranged from 21 to 25 (median = 22). Scores were highest within the product (re)formulation (median = 60) followed by the corporate nutrition strategy (median = 59) domain for manufacturers, while retailers performed best within the corporate nutrition strategy (median = 53), followed by the disclosure of relationships with external organizations (median = 47) domain. Companies within both sectors performed worst within the product accessibility domain (medians = 8 and 0 for manufacturers and retailers, respectively).

**Conclusions:**

This study highlights important limitations to self-regulatory approaches of the food and beverage industry to improve the healthfulness of food environments. Although some companies had specific, comprehensive, and transparent policies and commitments to address the healthfulness of food environments in Canada, most fell short of recommended best-practice. Additional mandatory government policies and regulations may be warranted to effectively transform Canadian food environments to promote healthier diets and prevent related non-communicable diseases.

**Supplementary Information:**

The online version contains supplementary material available at 10.1186/s12889-024-19864-1.

## Introduction

Current diets constitute an important risk for the development of non-communicable diseases (NCDs) across the globe. In 2017, it was estimated that 11 million global deaths were attributable to dietary risk factors, such as high intakes of sodium and low intakes of whole grains and fruits and vegetables [1]. In Canada, dietary risk factors are highly prevalent; 58% of Canadians aged 1 year and older have sodium intakes that exceed the recommended upper limit of 2300 mg/d [2], and 71% of Canadians aged 12 and older report consuming fruits and vegetables less than 5 times/day [3].

There have been several calls to action from the World Health Organization (WHO) and other public health interest groups identifying policies and actions that governments and food and beverage companies can address to promote healthier population diets and contribute to the prevention of diet-related NCDs [4–7]. These evidence-based policy and practice recommendations include reformulating products high in nutrients of public health concern (e.g., sodium, sugars, and saturated and trans fats), restricting the marketing of less healthy foods, increasing access to healthier foods, and providing easy to interpret nutrition information to consumers [6, 8].

Increasing the transparency and accountability of food and beverage companies for their role in the prevention of diet-related NCDs may contribute to improvements in their policies and commitments, and similarly draw attention to policy gaps where increased government regulations may be warranted [9]. Globally, a variety of approaches to independent monitoring of food and beverage company policies and action have been underway [10–14]. The *Business Impact Assessment – Obesity and population level nutrition* (BIA-Obesity) tool was developed by the *International Network for Food and Obesity / non-communicable Diseases Research, Monitoring and Action Support* (INFORMAS) to benchmark the food environment-related policies and commitments of food and beverage manufacturing, retailing and restaurant companies to support the prevention of obesity and other diet-related NCDs at a country level, beyond what is required by national legislation [15]. The BIA-Obesity tool evaluates a range of food environment action areas such as product (re)formulation for nutrients of concern, and product and brand promotion, among others. Methods were adapted from the *Access to Nutrition Index (ATNI),* a benchmarking initiative which originally assessed food and beverage manufacturing company policies related to all forms of malnutrition, including obesity and undernutrition, at a global level, with some national spotlights [10, 15]. To date, the BIA-Obesity tool has been used in New Zealand [16], Malaysia [17], Canada [18], Australia [19], Belgium [20], France [21], the European Union [22] and the United States [23].

Canada was one of the first countries to implement the tool to evaluate the food and beverage manufacturing sector in 2018 [18]. This study, which assessed the top 22 packaged food and non-alcoholic beverage manufacturers by market share in Canada, found that although most companies acknowledged their responsibility in addressing population health and nutrition, the specificity, comprehensiveness and transparency of commitments was inconsistent across the sector and there was considerable room for improvement across all areas [18]. The healthfulness of the products manufactured by these same companies was subsequently evaluated; most products were found to be less healthy, according to government-endorsed nutrient profiling models [24].

Since 2018, several policies and guidelines have been implemented or updated by the federal government to improve the healthfulness of food environments in Canada. This includes regulations for both back- and front-of-package nutrition labelling [25], a ban on industry use of partially hydrogenated oils (i.e. trans fats) in foods [26], and updated voluntary sodium reduction targets to guide the (re)formulation of processed foods [27].

That said, the healthfulness of Canadian food environments currently remains heavily reliant on food and beverage company voluntary policies and actions. For instance, despite two bills being introduced to restrict the advertising of less healthy food and beverage products to children and a proposal to regulate advertising to children under age 13 in Canada, advertising to children remains self-regulated by the food and beverage industry [28–30]. Since June 2023, the food and beverage industry has set forth the Code for the Responsible Advertising of Food and Beverage Products to Children in Canada (CCFBA) which restricts child-directed advertising of products that do not meet specific nutrition criteria, to those aged under 13 years [31–33].

Over the last few years, there has been increased scrutiny of the grocery retailing sector in Canada amidst rapidly rising food prices, competition concerns, as well as the development of a new grocery code of conduct (which aims to improve competitive dynamics between suppliers and retailers) [34, 35]. A small number of predominantly domestic retailers dominate the Canadian market [36], underscoring the relevance of evaluating the nutrition-related policies and commitments of leading companies to help understand how these powerful industry actors are contributing to the healthfulness of Canadian food environments.

Given recent changes in Canadian food environments and the important role of food and beverage companies in shaping the healthfulness of food environments, this study aimed to evaluate the voluntary policies and commitments of leading food and beverage manufacturers and grocery retailers to improve the healthfulness of population diets and prevent diet-related NCDs in Canada as of 2023, using the BIA-Obesity tool.

## Methods

The study adapted the BIA-Obesity tool developed by INFORMAS and first applied in Canada in 2018 to evaluate the specificity, comprehensiveness and transparency of voluntary policies and commitments of leading food and beverage manufacturing and retailing companies in Canada. Policies and commitments across 6 key action areas (hereafter referred to as ‘domains’) were assessed: 1) corporate nutrition strategy; 2) product (re)formulation; 3) nutrition labelling and information; 4) product and brand promotion including marketing to children and adolescents; 5) product accessibility including price, distribution and availability; and 6) disclosure of relationships with external organizations (see Table 1 for a description of each domain) [15].

**Table 1:** BIA-Obesity Canada 2023 domains, key indicator categories and weighting for each domain

Domain	Domain description	Key indicator categories	Weighting (%)	
			**Manufacturing**	**Retailing**
**Corporate nutrition strategy**	Overarching commitment to improving population nutrition for obesity and NCD prevention	– Commitment to nutrition and health in corporate strategy – Reporting against specific and measurable nutrition and health objectives and targets – Key Performance Indicators (KPIs) and remuneration of management linked to nutrition and health-related targets – Reporting on the percent sales volume of healthier product categories, and associated targets	10	10
**Product (re)formulation**	Commitment to addressing nutrients of concern (sodium, saturated fat, added/free sugars) and portion size in the development and reformulation of products	– Overarching commitment to addressing the healthfulness of products and brands, and targets related to sodium, free/added sugars, saturated fat, and portion size/energy density (where relevant) – Engagement with initiatives related to product formulation (e.g., Health Canada's voluntary 2025 sodium reduction targets)	30	25
**Nutrition labelling and information**	Commitment to providing comprehensive nutrition information across settings	– Provision of added sugar information – Provision of in-store^a^ and online nutrition information – Use of nutrient content claims	20	15
**Product and brand promotion**	Commitment to reducing the promotion of less healthy products on broadcast, digital and other non-broadcast media	– Policies restricting marketing to which children and adolescents are exposed on broadcast, digital and other non-broadcast media, and the use of marketing techniques that appeal to children and adolescents – Support for government policies restricting food and beverage marketing to which children and adolescents are exposed – Policies restricting less healthy product and brand promotion to the general population – Disclosure of marketing expenditures for healthier and less healthy foods and beverages to children, adolescents and adults	30	25
**Product accessibility**	Commitment towards addressing the availability, affordability and distribution of healthier and less healthy products	– Commitment towards addressing the availability (e.g., in schools) and distribution (e.g., placement within retail settings) of healthier and less healthy products – Commitment towards addressing the price of healthier and less healthy products – Support for government fiscal policies	5	20
**Disclosure of relationships with external organizations**	Policies and disclosures regarding funding or support for political parties, government agencies, professional organizations, external research, philanthropic groups, nutrition programs and active lifestyle programs	– Transparency of support for external organizations – Transparency of lobbying practices and political contributions	5	5

^a^Specific to grocery retailers

### Adapting the BIA-Obesity tool

First, the BIA-Obesity tool was adapted to the current Canadian policy context, including removal of indicators pertaining to government-mandated policy areas (i.e., where companies are obliged to meet indicator criteria). For instance, indicators related to the voluntary use of partially hydrogenated oils (or trans fats) were removed from the ‘product (re)formulation’ domain, as such additives are no longer permitted in food products sold in Canada since 2018 [26]. Similarly, indicators related to the voluntary use of front-of-package labelling, and associated support for the development of government policies in this area were removed from the ‘nutrition labelling and information’ domain. Indicators pertaining to the ‘product and brand promotion’ domain were also updated based on emerging best practice recommendations from the WHO [37], and additional indicators to assess marketing practices aimed at the general population were incorporated. Indicators were also updated to reflect overall progress in food industry strategies related to nutrition in consultation with the international INFORMAS team. For example, an indicator was added to assess if companies were reporting and publishing on the overall proportion of their sales volume from healthier and less healthy products and an associated target; in recent years, this metric has emerged as a priority indicator of a company’s overall performance on nutrition [38, 39]. The complete list of indicators and scoring criteria used in Canada in 2023 are available in Supplementary Table A1.

The final tool was composed of 6 domains (listed above and in Table 1), divided into 18 key indicator categories composed of 47 iscored ndicators for the food and beverage manufacturing sector and 64 scored indicators for the grocery retailing sector.

### Benchmarking company policies and commitments as of 2023

#### Selection of companies for assessment

Companies within the packaged food and non-alcoholic beverage manufacturing and grocery retailing sectors were selected based on the most recent market share data available on Passport, by Euromonitor International [40], and an analysis of the structure of the two aforementioned sectors in Canada [36]. Packaged food and non-alcoholic beverage manufacturing companies (hereafter referred to as ‘manufacturers’) were selected if they accounted for ≥ 1% of company market shares as of 2020 (packaged food) and 2021 (non-alcoholic beverage), and/or if they had been included in the BIA-Obesity study conducted in Canada in 2018. Two companies were excluded due to product portfolios of limited interest in the context of this analysis (i.e., bottled water and dry pasta manufacturers). The top 4 modern (chain) grocery retailing companies (hereafter referred to as ‘retailers’) as of 2021 were included, as these together accounted for over 80% of the sector in 2021 [40]. In the case that a company met the selection criteria for both manufacturers and retailers (e.g., Loblaw Companies Ltd), they were assessed independently for each sector (i.e., using both sets of indicators), and compared with manufacturers and retailers independently.

#### Data collection

Data related to the policies and commitments of selected companies were collected by AGH via publicly available sources including company websites, corporate social responsibility reports, industry association websites, and targeted online searches between August 2022 and June 2023. A secondary scan of company websites was conducted to ensure that the most recent corporate social responsibility reports (i.e., published by the end of the data collection period) were captured by the research team (if the company published such reports). All identified documents were downloaded, and webpages were saved by means of screenshots.

Company representatives were contacted between June and August 2023 via email, telephone, LinkedIn and/or company website correspondence pages and invited to participate in the study. Major industry associations in Canada were also contacted and invited to share information about this research with their members.

Companies that agreed to participate received a survey by e-mail with the compiled data for verification, and were given the opportunity to respond, correct errors, and supplement publicly available data by email, telephone, and/or videoconference. Information provided by companies was requested to be substantiated with appropriate documentation. At a company’s request, non-disclosure agreements were signed to ensure proprietary data would remain confidential, and solely used for assessment purposes (*n* = 2). The research team accepted company information until October 2023.

Companies were given a minimum of 3 weeks to complete the survey. Once received by the research team, completed surveys were reviewed. Companies were contacted again on a case-by-case basis for clarification purposes; no additional information beyond that required for clarification purposes was considered (e.g., if the company published a new policy document once the data validation and supplementation process had been completed, it was not included in the current research).

Ethics exemption was received from Université Laval (#2022–204).

#### Scoring of company policies and commitments

All indicators within each domain for each company were scored in Excel by AGH based on the specificity, comprehensiveness and transparency of policies and commitments (see Supplementary Table A1 for scoring criteria). A subsample of companies (4 manufacturers and 2 retailers) were also double-scored by a second researcher (LV). Discrepancies in scoring were discussed and resolved, in consultation with the broader international research team. Issues or adaptations to scoring practices identified during the double-scoring process were applied to the rest of the scoring.

Indicators which were not relevant to specific companies based on the composition of their product portfolio were considered as non-applicable, and scores were adjusted accordingly. For example, a company which solely manufactured sugary beverages would not have been expected to have (re)formulation targets for sodium, and related indicators would have been removed from the ‘product (re)formulation’ domain. If an indicator was removed, the total possible points within a domain were adjusted.

Each company was then given a score out of a total of 100 points for each domain. Domain scores were then weighted as described in Table 1, to yield a BIA-Obesity score out of a total of 100 points. Weights attributed to each domain were based on their relative importance (i.e., their potential impact) on obesity and population nutrition, as established by a group of food policy experts through several rounds of consultation, as described in a previously published paper [15]. Higher BIA-Obesity scores indicated stronger company policies and commitments with potential positive impacts on obesity and population nutrition.

Each company with an established contact was provided with a scorecard summarizing their results (overall and by domain) compared to other companies within their sector, highlighting areas in which they were demonstrating leadership, as well as priority areas for improvement. Summary sector reports were also created [13].

### Data analysis

Overall weighted and domain-specific median scores and ranges were assessed for each sector. Sensitivity analyses were conducted to examine differences in overall weighted scores between participating (i.e., companies that participated in the data verification and supplementation process) and non-participating companies to assess how the research process may have influenced final scores using Wilcoxon Rank Sum tests. Statistical analyses were conducted in SAS On Demand for Academics.

Leading policy and commitment examples among companies in the sample were identified for each domain and key indicator category. Examples were selected from companies across both sectors that obtained the highest scores within each domain and/or that demonstrated particular leadership within a key indicator category.

## Results

### Company participation

A total of 22 manufacturers and 4 retailers were assessed. These companies represented approximately 49% of the packaged food and 75% of the non-alcoholic beverage manufacturing sectors, and 82% of the chain grocery retailing sector. In total, 45% of manufacturers (*n* = 10) and 0% of retailers (*n* = 0) participated in the data verification and supplementation process. Two companies (i.e., Loblaw Companies Ltd and Sobeys Inc) were assessed as both manufacturers (of own-brand or private label products) and retailers.

### Overall results

**Fig. 1:**
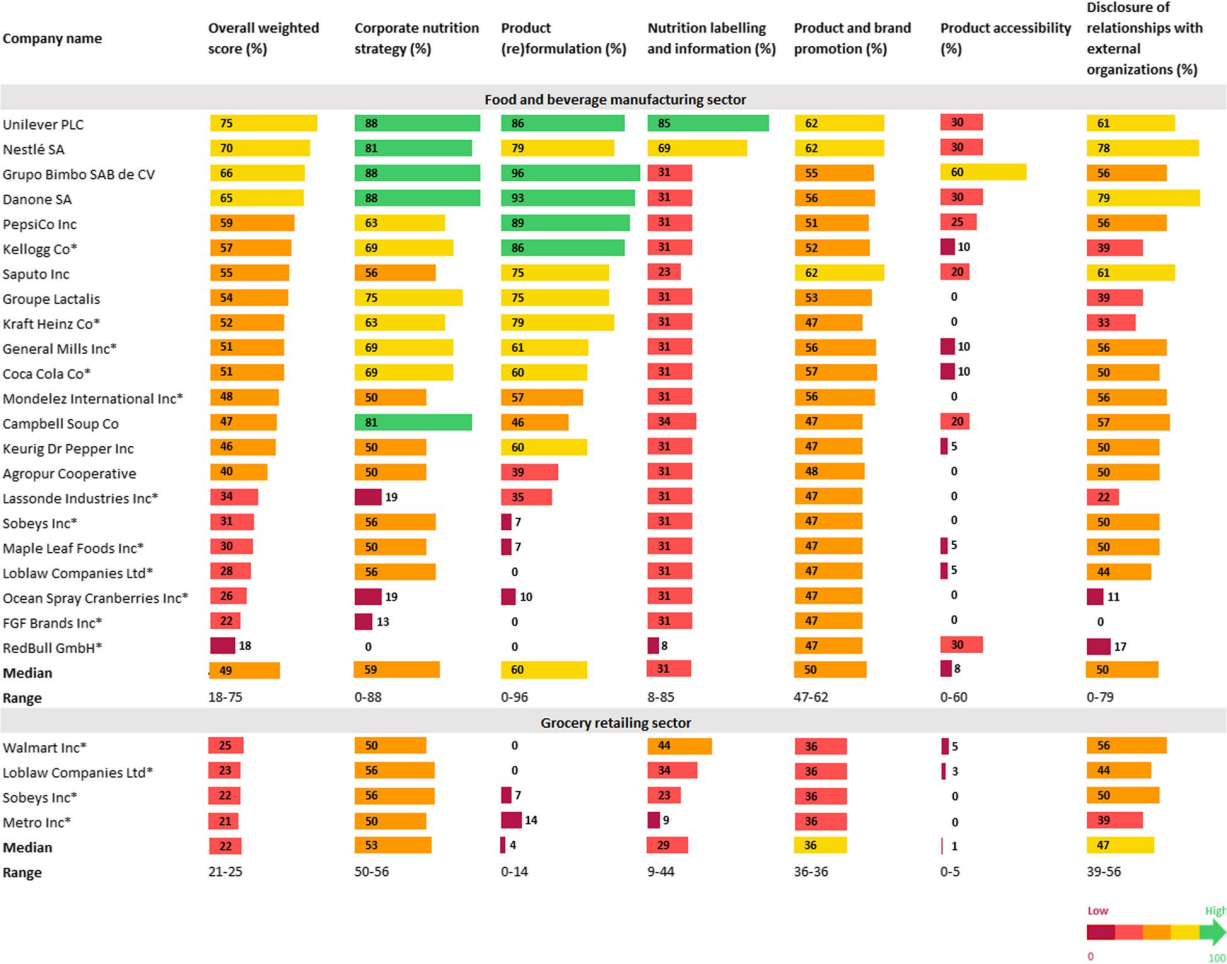
Overall weighted and domain-specific scores by company and sector. Overall weighted and domain-specific scores by company and sector are visually presented using a color code whereby scores 0-<20 are in burgundy, 20-<40 in red, 40-<60 in orange, 60-<80 in yellow and 80–100 in green. Higher scores indicate stronger policies and commitments. Companies that did not participate in the data validation and supplementation process are identified with an asterisk. All listed company names correspond with the name of the parent company. The name of Canadian subsidiaries are listed in a previously published market structure analysis [36]

Overall weighted and domain-specific scores by company and sector are presented in Fig. 1, and leading policy and commitment examples are listed in Table 2.

**Table 2:** Leading company policy and practice examples, by domain and key indicator category identified in Canada, as of 2023

Domain	Indicator category	Leading policy or practice examples
**Corporate nutrition strategy**	Commitment to nutrition and health in corporate strategy	– Unilever PLC had a clear commitment to tackling obesity, and a comprehensive set of nutrition-related objectives outlined in its Future Foods Positive Nutrition Action Plan, which explicitly applied to all markets. The company’s nutrition strategy referenced the United Nations Sustainable Development Goals, and the World Health Organization’s Action Plan for the Prevention and Control of Non-Communicable Diseases 2013–2020.
	Reporting against specific and measurable nutrition and health objectives and targets	– Danone SA published annual global reports and investor reports that included consolidated reporting on relevant nutrition-related topics, goals and associated progress. The company also reported on nutrition-related initiatives in Canada within national reports. – Grupo Bimbo SAB de CV published annual global reports with comprehensive reporting on nutrition-related topics and metrics, including national-level reporting on progress related to product reformulation, and partnerships. The company also reported on nutrition-related initiatives in Canada on its national website. – Nestlé SA published annual global reports with comprehensive reporting on nutrition-related topics and metrics, as well as national snapshots covering 2-year periods.
	Key Performance Indicators (KPIs) and remuneration of management linked to nutrition and health-related targets	– Kraft Heinz Co linked all Environmental, Social and Governance (ESG) initiatives to CEO and leadership compensation.
	Reporting on the percent sales volume of healthier products (relative to overall or less healthy product sales), and associated targets	– Danone SA reported on the healthfulness of its product portfolio using various definitions of ‘healthy’, including government-endorsed systems (Health Star Rating and Nutri-Score). The company had many targets to increase the percent sales volume of healthier products, such as a target for at least 85% of volumes sold of dairy, plant-based, water and ‘aquadrinks’ to have a Health Star Rating of at least 3.5 by 2025. – Unilever PLC reported on the percent sales volume of healthier products using various definitions of ‘healthy’, including government-endorsed systems and the Unilever Science-based Nutrition Criteria (USNC). The company set a target for having 85% of servings sold meet the USNC by 2028.
**Product formulation**	Overarching commitment to addressing the healthfulness of products and brands, and targets related to sodium, free/added sugars, saturated fat, and portion size/energy density (where relevant)	– Up to 2022, Grupo Bimbo SAB de CV had specific, measurable, achievable, relevant and time-bound (SMART) targets to reach thresholds for nutrients of public health concern for products for daily consumption, and nutritional guidelines for products for occasional consumption. Reporting on progress overall and for each nutrient was made publicly available in the global ESG report, including national level data. The Grupo Bimbo strategy evolved to include SMART targets, based on Health Star Rating thresholds, guiding the reformulation of products for daily and occasional consumption, for both 2025 and 2030. – PepsiCo Inc had SMART targets to reduce added sugars in beverages, and sodium and saturated fats in foods by 2025, with a 2020 baseline. PepsiCo Nutrition Criteria, the company’s nutritional profiling model used to guide product development, has been published in peer-reviewed literature. The company publicly reported on progress, on an annual basis at the global level, with 3rd party limited assurance. – Unilever PLC reported on past progress for production (re)formulation and had SMART forward-looking targets. The company had a target for 70% of products to meet their WHO-aligned targets by 2022; in 2022, 64% of products met the publicly available nutritional criteria which included thresholds for calories, sugar, sodium and saturated fat. This commitment was replaced with a target for 85% of the company’s portfolio to meet the USNC across all product groups by 2028. Evidence behind the development of the company’s (re)formulation criteria has been published in the peer-reviewed literature.
	Engagement with initiatives related to product formulation (e.g., Health Canada's 2025 sodium reduction targets)	– Groupe Lactalis (specifically, Lactalis Canada) had a public commitment to meet Health Canada’s sodium reduction targets for cottage cheese, processed cheese, natural cheese and butter and was in the process of assessing its portfolio against Health Canada’s front-of-package labelling thresholds for sugar and saturated fat.
**Nutrition labelling and information**	Provision of added sugar information	– Campbell Soup Company (specifically, The Campbell Company of Canada) provided information on added sugar for some products online.
	Provision of in-store and online nutrition information	– Keurig Dr Pepper Inc provided complete nutrition information online in a user-friendly format. On top of information available for pre-packaged beverages, fountain beverages could be selected, with customization possible, including the proportion of the drink which is composed of ice cubes. – Walmart Inc provided comprehensive online nutrition information in the form of nutrition facts tables. Walmart also provided filters which shoppers could select based on their lifestyle and dietary needs, such as ‘no added sugars’
	Use of nutrient content claims	– Danone SA committed to not displaying nutrition or health claims on products with a Health Star Rating below 2.5 stars, with a transition period taking place until October 2024.^a^
**Product and brand promotion**	Policies restricting marketing to which children are exposed on broadcast, digital and other non-broadcast media, and the use of marketing techniques that appeal to children and adolescents	– Saputo Inc restricted marketing to children under the age of 15 years on all media (with an audience threshold of 30%) and settings, including packaging and point-of sale marketing. Saputo committed to only market products that meet their level 1 (most stringent) publicly available nutrition criteria to children, and not market butter, cream and ice cream to children. – Nestlé SA restricted marketing to children under 16 on TV and online media (with an audience threshold of 25%). The policy applied nutrition criteria determined by nationally or regionally agreed-upon pledges, and prohibited direct advertising of confectionery, ice cream and water-based beverages with added sugars to children under 16. – Unilever PLC restricted marketing to children under the age of 16 on traditional and digital media (with an audience threshold of 25%). Different nutrition criteria apply depending on the media or setting (i.e., all products, Highest Nutritional Standards or Responsibly Made for Kids).
	Support for government policies restricting food and beverage marketing to which children are exposed	– *No leading practice examples were identified*
	Policies restricting less healthy product and brand promotion to the general population	– Agropur Cooperative committed to the responsible promotion of its products in alignment with Health Canada and United States Department of Agriculture nutrition recommendations
	Disclosure of marketing expenditures for healthier and less healthy foods and beverages to children, adolescents and adults	– Nestlé SA committed to increasing the proportion of their marketing spend to promote healthier choices. In 2020, the company reported a 106% increase in marketing spend dedicated to products that support healthier lifestyles; however, this commitment was not relative to overall or less healthy product marketing
**Product accessibility**	Commitment towards addressing the availability and distribution of healthier and less healthy products	– Grupo Bimbo SAB de CV aimed to launch ≥ 1 program/region to support vulnerable populations through accessible (products with a wide-distribution range) and affordable (price ≥ 5% under the category average) products with positive nutrition by 2025. Positive nutrition would be defined using the Health Star Rating as of 2025. – Nestlé SA committed to only selling products that meet the Nestlé Marketing Communication to Children Policy Nutrition Criteria in primary and secondary schools.
	Commitment towards addressing the price of healthier and less healthy products	– *See above for commitment made by Grupo Bimbo.*
	Support for government fiscal policies	– *No leading practice example identified.*
**Disclosure of relationships with external organizations**	Transparency of support for external organizations	– Nestlé SA disclosed support provided to community/philanthropic organizations in a consolidated manner within national reports. The company also disclosed public private partnerships, and industry partners in a consolidated way on its global website. – Danone SA disclosed community engagement-related goals and achievements in a consolidated manner within a table in national reports. Collaborations with professional organizations to promote health education were also consolidated within the report. Project grants provided by the Danone Institute North America were consolidated and described online. – The PepsiCo Foundation (through which most of PepsiCo Inc’s philanthropic efforts are directed) consolidated its support for philanthropic groups within a publicly available document, which included the name of the organization supported, amount donated, program, focus area and beneficiary country. The PepsiCo Health and Nutrition Sciences team published its research on its website in a consolidated manner, by topic area. – Unilever PLC published a consolidated list of nutrition publications (2009–2022) supported by the company. – Walmart Inc provided a consolidated and interactive list of organizations which received funding (grants over $25 000) from Walmart and the Walmart Foundation. Walmart also published a consolidated list of support for community organizations specific to Canada.
	Transparency of lobbying practices and political contributions	– Nestlé SA publicly committed to not make political contributions, with the exception of the parent company, within its country of origin. – Unilever PLC publicly committed to not support or make political contributions to political parties, candidates or groups endorsing party interests.

^a^This policy was published after Danone’s data collection and was not considered in their score

Overall weighted scores ranged from 18 to 75 points out of 100 (median = 49) for manufacturers, and 21 to 25 out of 100 (median = 22) for retailers. Manufacturers performed best within the ‘product (re)formulation’ (median = 60) and ‘corporate nutrition strategy’ (median = 59) domains, while retailers performed best within the ‘corporate nutrition strategy’ (median = 53) and ‘disclosure of relationships with external organizations and lobbying’ (median = 47) domains. Manufacturers and retailers both performed worst within the ‘product accessibility’ domain (medians = 8 and 0, respectively).

There was strong evidence of a difference in the scores of participating (*n* = 10, median = 57) and non-participating manufacturers (*n* = 12, median = 32) (*p* = 0.003). Differences between groups were not evaluated for retailers as none participated in the data verification and supplementation process.

### Results by domain

#### Corporate nutrition strategy

Scores (out of 100) within the ‘corporate nutrition strategy’ domain ranged from 0 to 88 (median = 59) for manufacturers, and 50 to 56 (median = 53) for retailers. Most manufacturers (*n* = 21/22) and all retailers (*n* = 4/4) made some mention of nutrition and health within their corporate strategy. Most companies across both sectors (*n* = 18/22 manufacturers and *n* = 4/4 retailers) had national and/or global reporting of corporate social responsibility initiatives, with varying levels of reporting on nutrition-related policies, objectives and targets. A total of 6/22 manufacturers and 0/4 retailers explicitly linked performance against nutrition-related metrics to executive/leadership compensation.

A total of 8/22 manufacturers and 0/4 retailers reported on the sales volume of healthier products. Of these, 5 companies used at least 1 government-endorsed nutrient profiling model to define ‘healthier’ foods for their reporting, and 2 companies reported on targets to increase the percentage (or proportion) of their sales volume of healthier products.

#### Product (re)formulation

Scores (out of 100) within the ‘product (re)formulation’ domain ranged from 0 to 96 (median = 60) for manufacturers, and 0 to 14 (median = 4) for retailers. A total of 16/22 manufacturers and 1/4 retailers had an overarching commitment related to improving the nutritional composition of their product portfolio; 13/17 manufacturers had sodium targets/guidelines, while 11/17 had targets/guidelines for saturated fats, 15/22 for sugars and 12/22 for energy/portion size. A total of 4/22 manufacturers published their nutrient profiling model or scientific basis behind their classification system in the peer-reviewed literature, and 4/22 used a government-endorsed classification system covering nutrients of public health concern (e.g., Health Canada’s front-of-packaging labelling thresholds [25], the Health Star Rating system which assesses both positive and negative nutrients [41] or others). No retailers had published targets or guidelines for the aforementioned nutrients of public health concern.

#### Nutrition labelling and information

Scores (out of 100) within the ‘nutrition labelling and information’ domain ranged from 8 to 85 (median = 31) for manufacturers, and 9 to 44 out of 100 (median = 29) for retailers.

The majority of manufacturers (*n* = 21/22) and retailers (*n* = 3/4) provided comprehensive online nutrition information for most or all products/brands. Most retailers (*n* = 3/4) also had some strategies to guide consumer purchases to identify healthier foods in online settings (e.g., via product filters to select healthier foods); however, few in-store strategies were identified (e.g., use of shelf-tags with summary nutrition information, or ongoing in-store healthy eating education programs). Only one company across both sectors (The Campbell Soup Company) was found to provide information for added sugars for some products online.

Few companies (manufacturers: 2/22, retailers: 0/4) pledged that nutrition and health claims would not be displayed on less healthy products, such as those that will be required to carry a front-of-package nutrition symbol in Canada. Companies with commitments (Nestlé SA and Unilever PLC) used company-developed nutrient profiling models to define the healthfulness of products that were permitted to carry nutrition and health claims.

#### Product and brand promotion

Scores (out of 100) within the ‘product and brand promotion’ domain ranged from 47 to 62 (median = 50) for manufacturers, and all retailers obtained a score of 36.

All companies across both sectors restricted some child-directed advertising via the new industry Code for the Responsible Advertising of Food and Beverage Products to Children in Canada (CCFBA). A total of 10/22 manufacturers and 0/4 retailers had company-specific policies beyond the CCFBA. Of these, 3/22 restricted marketing using an age threshold above 13 years, and 8/22 restricted certain child-directed or child-appealing marketing techniques on product packaging. No company, across both sectors, publicly supported government policies to restrict the marketing of less healthy food and beverages to children, as recommended by the WHO [37].

Few commitments regarding restricting less healthy product and brand promotions to the general (adult) population were identified across both sectors. No retailers had policies restricting in-store promotions of less healthy products or ensuring in-store product presentation, giveaways and tastings were for healthier products, increasing the proportion of healthier foods promoted in catalogues/flyers, or promoting healthier purchases via rewards and loyalty programs.

Across both sectors, no company had targets and associated reporting on the proportion of marketing expenses dedicated to healthier products.

#### Product accessibility

Scores (out of 100) within the ‘product accessibility’ domain ranged from 0 to 60 (median = 8) for manufacturers, and 0 to 5 (median = 1) for retailers.

Few companies across both sectors had any type of commitment or policy to address the availability and distribution of healthier products relative to less healthy products in different settings (e.g., schools, hospitals, remote communities, and community spaces) or for retailers, in high-traffic locations (e.g., checkouts and end-of-aisle displays). Most commonly, policies were related to regulating the availability of healthier and less healthy products in schools (*n* = 4/22 manufacturers).

Similarly, few companies across both sectors had any type of commitment or policy to address the price of healthier products relative to less healthy products. Grupo Bimbo SAB de CV was the only company to have a specific, time-bound target to address the price of healthier products (see Table 2).

No company published a statement in support of WHO-recommended fiscal policies to make healthier products relatively less expensive and/or less healthy products relatively more expensive, such as a tax on sugary beverages.

#### Disclosure of relationships with external organizations

Scores (out of 100) within the ‘disclosure of relationships with external organizations’ domain ranged from 0 to 79 (median = 50) for manufacturers, and 39 to 56 (median = 47) for retailers. Most companies across both sectors (manufacturers: 17/22, retailers: 4/4) disclosed consolidated and national-level information on philanthropic activities and support for community organizations. The disclosure of consolidated and nationally representative information pertaining to support and funding of research, scientific events, professional organizations, public–private partnerships, and industry associations was less pervasive across companies in both sectors.

A total of 8/22 manufacturers and 0/4 retailers had a policy regarding the disclosure of political contributions, or a commitment to not making political contributions in Canada. No company across both sectors had a commitment to disclosing their submissions to public consultations on topics related to nutrition beyond legal requirements.

## Discussion

This study assessed the specificity, comprehensiveness and transparency of the nutrition-related policies and commitments of the largest 22 food and beverage manufacturers and 4 grocery retailers in Canada as of 2023. Stronger policies and commitments were observed among manufacturers (median = 49 out of 100, range = 18 to 75) compared to retailers (median = 22 out of 100, range = 21 to 25). Within the manufacturing sector, relatively high scores (≥60) by at least one company within each domain reflects the potential for companies to adopt strong food environment policies and commitments. There were large discrepancies both within and between companies, and most companies, across both sectors, fell short of recommended best practice policies and commitments to support healthier food environments in Canada.

### Policies and commitments across domains and sectors as of 2023

As of 2023, companies within the manufacturing sector performed best within the ‘product (re)formulation’ and ‘corporate nutrition strategy’ domains, while retailers also performed best within the ‘corporate nutrition strategy’ domain. These results suggest that companies are acknowledging their important role in contributing to the healthfulness of the diets of the population; however, results within other domains demonstrate that this overarching commitment to population nutrition is most often not supported by a comprehensive set of policies and commitments across key food environment action areas. This aligns with findings from previous assessments conducted across the globe; ‘corporate nutrition strategy’ has been the first or second highest scoring domain in most countries where the BIA-Obesity tool has been implemented for manufacturers [16–22] and/or retailers [16, 19–22], including Malaysia (2017), New Zealand (2017), Canada (2017–18), Australia (2018), Belgium (2019–20), France (2019–20), and the European Union (2020). This also aligns with the ATNI Global Index 2021 which found ‘governance’ to be the highest scoring of 7 domains among the top 25 global food and beverage manufacturers [42].

Retailers performed poorly within the product (re)formulation domain compared to manufacturers. This may be indicative of a lack of action from retailers, and/or a lack of transparency regarding how retailers are shaping the nutritional quality of the food supply in Canada. This may be due to competitive concerns, and/or limited resource as no company participated in the data validation and supplementation process. Own-brand (or private label) products manufactured by leading retailers are among top selling products within several product markets in Canada [36] and thus represent an important contribution to Canadian dietary patterns, and greater transparency is needed in related manufacturing practices.

Although ‘product (re)formulation’ was among the highest scoring domains for manufacturers, many companies in this sector did not have published targets related to nutrients of public health concern; 6/22 companies obtained very poor scores (≤ 10 out of 100). Moreover, very few companies explicitly aligned reformulation efforts with government- or WHO- endorsed nutrient profiling models or targets. This study did not assess the strength or validity of industry-developed nutrient profiling models or compare them to existing government-endorsed systems. However, the use of government-endorsed systems increases the likelihood of these systems going through rigorous independent peer-review and scrutiny, transparency in development and validity, and increases comparison and accountability across companies, and thus is preferable to the use of industry-developed systems which may not have these same qualities. Previous monitoring of the product portfolios of the top 22 manufacturing companies in Canada (similar to those assessed in this study) showed that the mean healthfulness of product portfolios, measured using the Health Star Rating, had improved among 5 companies and worsened for 1 company between 2013 and 2017; however, little change was observed in the healthfulness of matched products over time [43]. At a global level, ATNI found that 9 out of 18 of the largest food and beverage companies had improved the sales-weighted mean Health Star Rating of their portfolio between 2018 and 2021 [44]. Monitoring the nutritional quality of the foods manufactured by companies included in this analysis will help understand if commitments are leading to meaningful (re)formulation actions in Canada.

Most companies across both sectors obtained poor scores within the ‘nutrition labelling and information’ domain. This can be explained by most companies not having policies to restrict the use of nutrition claims on less healthy products (e.g., those that will be required to carry a front-of-package symbol), and the larger weight attributed to this key indicator category due to the removal of indicators pertaining to front-of-package labelling.

Companies across both sectors obtained relatively poor, but similar, scores within the ‘product and brand promotion’ domain, in part, due to the industry-wide code to restrict child-directed advertising (i.e., the CCFBA) [45]. Although the CCFBA has not been evaluated for its impact, voluntary industry efforts to restrict marketing to children in Canada as well as in other countries have, to date, been insufficient to adequately mitigate child and adolescent exposure to less healthy food marketing [46, 47]. The CCFBA represents a potential improvement over its predecessor (i.e., the Canadian Children’s Food and Beverage Advertising Initiative or CAI) as it includes a lower child audience threshold for measurable media such as TV broadcast (25–35% vs 15%), nutrition criteria that mostly aligns with Health Canada nutrient thresholds for foods ‘low in’ sodium, sugars and saturated fat, and applies to a greater number of companies [31, 33, 48, 49]. However, this voluntary Code falls short of recent recommendations for effectively reducing children’s exposure to unhealthy food marketing [37]. In particular, it does not restrict the use of brand marketing and sponsorship, its definition of advertising ‘directed to children’ is quite narrow, it excludes product packaging and social media not primarily directed to children from restrictions, and only protects children under age 13 whereas the recent WHO recommendation is to protect children up to age 18 [37]. Compliance with the CCFBA is also intended to be evaluated using a pre-screening process; however, there are no stated penalties for non-compliance [31].

No retailers reported having any type of policies or commitments within the ‘product and brand promotion’ domain beyond the CCFBA, such as limits on social media marketing (e.g., on Facebook, Instagram), limits on in-store promotions of less healthy products, increasing the proportion of healthier products promoted in flyers, and linking rewards/loyalty programs to healthier food items. In the global context, there are multiple examples of retailers that have implemented such policies [39, 50]. For instance, in Belgium, the retailer Delhaize has incentivized the purchase of healthier products, defined according to a government-endorsed nutrient profiling model (i.e., Nutri-Score A or B), with an automatic 5–15% discount via its loyalty program [50]. Policies to promote healthier product purchases may be particularly relevant in Canada where 75% of the population reports having a loyalty or points card from a grocery store and 62% indicates being more likely to buy from a store where they have such a card [34]. Moreover, 60% of products promoted in flyers have been found not to align with Canada’s Food Guide recommendations [51].

The ‘product accessibility’ domain was the worst performing domain across both sectors; it has also been found to be the worst, or second worst in terms of performance in almost all other countries that have implemented the BIA-Obesity tool [16–22], as well as the ATNI 2021 Global index [42]. Over the past 2 years, food inflation has been substantially higher compared to already elevated overall inflation rates in Canada [34, 52]. Although many factors have contributed to rising food prices, including the COVID-19 pandemic, climate crises and geopolitical conflicts which have resulted in supply chain disruptions [53, 54], innovation is needed to identify ways that companies can meaningfully contribute to improving the price, distribution, placement and availability of healthier foods in existing Canadian food environments. Some innovative global examples have been identified in other studies [50]. For instance, Outback Stores in remote Australian communities have committed to placing fruits, vegetables, and water fridges at the front of stores and sugary beverages at the back of stores, and to having at least 50% of displays made up of water and diet beverages [50, 55]. The company has also committed to pricing water at a lower price than diet and full sugar beverages.

### Changes in company policies and commitments over time in Canada

This study repeated a similar analysis of the policies and commitments of food and beverage manufacturing companies in Canada conducted in 2018 [18]. Direct comparisons were not conducted due to changes in indicators and scoring criteria made to reflect the evolving Canadian policy context and evidence-based recommended best-practice, as well as some changes to the structure of companies (e.g. George Weston Ltd, evaluated in 2018, sold its bread operations to FGF Brands Inc, evaluated in 2023).

Noteworthy changes over time within the manufacturing sector included stronger/more nutrition-related policies and commitments reflected by higher overall scores in 2023 (median = 49, range = 18 to 75) compared to 2018 (median = 27, range = 4 to 60). Sector-wide improvements were largest within the ‘product and brand promotion’ domain, mainly due to the industry-wide CCFBA, with the median score within this domain reaching 50 (range = 47 to 62) in 2023 compared to 32 (range = 0 to 51) in 2018. Given the heavier weighting of this domain in the overall score, sector-wide increases in these scores heavily influenced overall BIA-Obesity scores. Companies continued to perform relatively well within the ‘corporate nutrition strategy’ domain, which was the highest performing domain in 2018 (median = 63, range = 0 to 93) and second best in 2023 (median = 59, range = 0 to 88). That said, companies fell short of recommended best-practice in several similar areas in 2018 and 2023, in particular within the ‘product accessibility’ domain, where the median score was 8 (range = 0 to 60) in 2023 and 0 (range = 0 to 40) in 2018. These changes over time highlight some sector-wide improvements, as well as important remaining gaps.

### Strengths and limitations

This study evaluated a wide variety of nutrition-related policies and commitments and covered a large proportion of the Canadian food and beverage manufacturing and retailing sectors in Canada using internationally recognized methods, adapted to the Canadian policy context, which have been proven to have some positive effects in stimulating changes within companies [9].

There are several limitations to this study. First, there was a low participation rate, particularly within the retailing sector, which limited those company assessments to publicly available data. A statistically significant difference was observed between the scores of participating and non-participating manufacturers (as has been the case in BIA-Obesity assessments conducted in other countries [16, 17, 20, 21]), which indicates that the current study may underestimate the scores of companies that did not participate. Lack of participation may be due to limited resources to dedicate to these types of projects within companies. This nonetheless reflects a lack transparency around important nutrition topics, an aspect evaluated with the BIA-Obesity tool. Next, this study did not account for company practices. Future investigations could evaluate company performance and their compliance with commitments, for example in improving the healthfulness of their product portfolios or restricting their marketing practices of less healthy foods to which children may be exposed. Future work may also assess policies, commitments and actions related to environmental sustainability, as healthy and sustainable diets have similar causes and potentially synergistic mitigating actions [56]; INFORMAS has developed a tool which could be used as a starting point, in conjunction with the BIA-Obesity tool [57].

### Policy implications

There are numerous opportunities for companies to more actively contribute to healthier food environments. The first key opportunity is to improve reporting practices and national-level disclosures; transparency is necessary for public accountability [4]. The second opportunity is for companies to use government-endorsed nutrient profiling models for reporting and as a basis for (re)formulation and other targets. A wide variety of nutrient profiling models have been endorsed by governments worldwide [58]. In international benchmarking activities, nutrient profiling models that assess the global nutritional quality have typically been used, including the Health Star Rating system. In the Canadian context, using the front-of-package labelling cut-offs for foods high in sodium, sugar and saturated fat and/or the nutrient criteria for foods ‘low in’ these nutrients could support policy cohesion. Thirdly, companies have the opportunity to actively support government policies that aim to create healthier food environments. Mandatory policies that apply industry-wide create a level playing field for all companies, and do not rely on individual companies to act, nor investor or stakeholder approval. This is of particular importance as evidence indicates that mandatory policies have larger benefits for population health than voluntary or industry-led policies [59, 60]. In addition, studies in Canada have demonstrated that industry players are actively lobbying on topics related to nutrition in Canada, using a variety of persuasive techniques that are known to weaken or stymie mandatory public health policy [61–63].

In the absence of action from many companies, these results highlight opportunities for additional government intervention. This may include regulating corporate reporting regarding nutrition, such as on the percentage sales volume of products meeting a government-endorsed nutrient profiling model. Additional food environment policies including mandatory reformulation targets, restrictions on marketing to children (protecting all those < 18 years), nutrition standards for foods that can be sold in schools or public settings, as well as restrictions on product placements and discounts could ensure that all food companies act within these respective policy domains.

## Conclusion

The varied scores across domains and companies within the manufacturing sector and low scores across the retailing sector highlights policy gaps and opportunities to improve the healthfulness of Canadian food environments. The high scores obtained by at least one company within each domain, particularly within the manufacturing sector, demonstrates the feasibility of having specific, transparent and comprehensive nutrition-related policies and commitments, and the importance of continued monitoring of both policies and actual practices within food environments. The poor performance of many companies across both sectors supports previous research documenting the limitations of industry self-regulation [16, 17, 19, 20, 43, 64, 65]. Governments may create a level playing field for companies to effectively support healthier population diets by implementing robust and mandatory food environment policies.

## Supplementary Information

Supplementary Material 1.

## Data Availability

Data collated by the research team are available upon request, with the exception of limited information pertaining to 2 companies covered by a nondisclosure agreement. For more information, please contact Lana Vanderlee, at lana.vanderlee@fsaa.ulaval.ca

## Abbreviations

ATNI: Access to nutrition index
BIA-Obesity: Business impact assessment - Obesity and population level nutrition
CCFBA: Code for the responsible advertising of food and beverage products to children in Canada
ESG: Environmental, social and governance
INFORMAS: International network for food and obesity/non-communicable disease research, monitoring and action support
NCD: Non-communicable disease
USNC: Unilever science-based nutrition criteria
WHO: World health organization

## Data availability

Data collated by the research team is available upon request, with the exception of limited information covered by nondisclosure agreements. For more information, please contact Lana Vanderlee at lana.vanderlee@fsaa.ulaval.ca.
