# Supplementary material for: Food and beverage manufacturing and retailing company policies and commitments to improve the healthfulness of Canadian food environments
Source: BMC Public Health. 2024 Sep 5;24:2419. doi: 10.1186/s12889-024-19864-1 (PMC11375943; doi:10.1186/s12889-024-19864-1)
Supplement: Supplementary file 1 — Supplementary Material 1. [file 12889_2024_19864_MOESM1_ESM.docx]

**Supplementary Table A1**. Master list of indicators and scoring criteria for BIA-Obesity Canada 2023 for each sector and domain

| **Indicator identifier** | | **Indicator** | **Max points** | **Scoring criteria** |
| --- | --- | --- | --- | --- |
| **Manufacturing sector** | **Retailing sector** |  |  |  |
| **Corporate nutrition strategy (STRAT)** | | | | |
| M-STRAT1 | S-STRAT1 | Does the company have an **overarching commitment to population nutrition and health** articulated in strategic documents (e.g., mission statement, strategies, or overarching policies)? | 10 | 10: Yes, a specific national-level (country-specific) commitment to improving population nutrition and health, publicly available in strategic documents  7.5: Yes, a specific global-level (not country -specific) commitment to improving population nutrition and health, publicly available in strategic documents  5: Yes, a national- or global-level commitment, but not publicly available, OR general reference to nutrition and health as part of general corporate strategy  0: No clear commitments to improving population nutrition and health |
| M-STRAT2a | S-STRAT2a | Does the company’s commitment to improving population nutrition and health (where it exists) specifically mention obesity and NCD prevention? (include references to WHO/SDGs/other recognized reporting standards) | 5 | 5: Yes, commitment specifically refers to obesity and NCD prevention priority areas, as aligned with or as outlined in key government policy or international documents (for example the WHO Global Action Plan for the Prevention and Control of NCDs 2013-2020, Sustainable Development Goals, the WHO Report on Ending Childhood Obesity, and/or Health Canada's Healthy Eating Strategy) 0: Commitment does not refer to obesity and NCD prevention  * STRAT2a and STRAT2b were scored together (total possible points = 5). |
| M-STRAT2b | S-STRAT2b | Does the company’s commitment to improving population nutrition and health (where it exists) recognize or reference relevant priorities in Health Canada’s policy documents relating to population nutrition and obesity/NCD prevention? |  |  |
| M-STRAT3 | S-STRAT3 | Are Key Performance Indicators (KPIs) (and/or remuneration) of senior managers linked to population nutrition and health strategies/policies/targets? | 5 | 5: Key Performance Indicators (KPIs) (and/or remuneration) of senior managers are linked to nutrition strategy/policy/targets 2.5: KPIs (and/or remuneration) of lower management are linked to nutrition OR KPIs (and/or remuneration) of management are linked to nutrition topics, but not specifically topics that address nutrition-related NCDs 0: No evidence of how KPIs (and/or remuneration) are linked to nutrition and health targets |
| M-STRAT4 | S-STRAT4 | Does the company report how it progressing in achieving population nutrition or health objectives / targets? How often is this information updated? Is this publication audited and made available to the public? | 10 | 10: Regular, publicly available national reports including reporting against objectives and targets, a clear outlook of future plans and challenges, external verification / review, and specifically relate to the country in question  7.5: Regular, publicly available global reports that are not clear whether or not statistics within the report include the country in question  5: Annual reports including some of the relevant information  2.5: Irregular reporting  0: None published |
| M-STRAT5 | S-STRAT5 | Does the company publish a target and have associated reporting on the overall proportion of sales volume from healthy and unhealthy products? | 10 | 10: Yes, published target and regular reporting on the overall proportion of sales volume from healthy and unhealthy products, with product healthiness defined by government guidelines/government endorsed classification system  5: Yes, regular reporting on the overall proportion of sales volume from healthy and unhealthy products, but target is not published / only applies to some product categories / does not use government guidelines/government endorsed classification system to define product healthiness  2.5: Vague reporting on this topic  0: No information or no target |
| **Product (re)formulation (FORM)** | | | | |
| M-FORM1 | S-FORM1 | Does the company have a set of commitments or objectives related to new product development and reformulating its existing products (sodium, saturated fat, free/added sugars, caloric density/portion size)? Are these commitments published and publicly available? | 10 | 10: Yes, specific national-level commitments/objectives that are publicly available or specific global commitments/objectives that include specific reference to the country or market in question  7.5: Yes, specific global commitments/objectives that could specifically apply to the country in question that are publicly available  5: Has specific national-level commitments/objectives, but not publicly available  2.5: Has national or global-level commitments/objectives in this area that are available publicly, but these commitments/objectives are vague and non-specific  0: No |
| M-FORM2 | S-FORM2 | Is the company committed to reaching Health Canada's voluntary 2025 sodium reduction targets, or do they commit to other **initiatives on product reformulation**? | 10 | 10: Yes, government-endorsed or WHO targets, noted on company website or in annual reports  7.5: Yes, industry initiative, noted on company website or in annual reports  5: Yes, but NOT noted on company website/annual reports (e.g. government/ NGO/industry organisation’s website or disclosed directly to INFORMAS)  0: No  (*Full points were awarded to beverage companies for whom there are no government-endorsed sugar reduction targets in the Canadian market that publicly commit to an industry reformulation initiative). |
| M-FORM3 | S-FORM3 | **Salt/sodium**: Has the company set a target/targets to reduce salt/sodium in their products? If so, what are the targets and the baseline year/target year? | 10 | 10: Set SMART targets or provided detailed evidence of having taken significant action in all key categories/subcategories, published^1^  5: Targets (not necessarily SMART) set or significant action taken in some key products/sub-categories / not published  2.5: General commitment to reducing levels of salt/sodium in products (vague or global level only), published or disclosed to INFORMAS team  0: No target / no information |
| M-FORM4 | S-FORM4 | **Saturated fat**: Has the company set a target/targets to reduce levels of saturated fat in their products? If so, what are the targets and the baseline year/target year? | 10 | 10: Set SMART targets or provided detailed evidence of having taken significant action in all key categories/subcategories, published  5: Targets (not necessarily SMART) set or significant action taken in some key products/sub-categories / not published  2.5: General commitment to reducing levels of saturated fat in products (vague or global level only), published or disclosed to INFORMAS team  0: No target / no information |
| M-FORM5 | S-FORM5 | **Sugars** (free or added): Has the company set a target/targets to reduce sugars in their products? If so, what are the targets and the baseline year/target year? | 10 | 10: Set SMART targets or provided detailed evidence of having taken significant action in all key categories/subcategories, published  5: Targets (not necessarily SMART) set or significant action taken in some key products/sub-categories / not published  2.5: General commitment to reducing levels of sugar in products (vague or global level only), published or disclosed to INFORMAS team  0: No target / no information  ****for water-based beverage-only companies*** 5/10 if the company has SMART kcal targets, but NO SMART target for free/added sugars* |
| M-FORM6 | S-FORM6 | **Portion size/energy content**: Has the company set a target/targets to reduce the portion size or energy content per serving of products/in single serve snacks? If so, what are the targets and the baseline year/target year? | 10 | 10: Set SMART targets or provided detailed evidence of having taken significant action in all key categories/subcategories, published  5: Targets (not necessarily SMART) set or significant action taken in some key products/sub-categories / not published  2.5: General commitment to reducing levels of calories in products (vague or global level only), published or disclosed to INFORMAS team  0: No target / no information |
| M-FORM7.1 | S-FORM7.1 | What system / criteria (e.g., product classification system or nutrient profiling system) does the company use to classify the healthiness of products for the purposes of product development / reformulation? | 10 | 10: Uses government guidelines/government endorsed classification system (where available)  7.5: Publicly available system, developed in consultation with experts and in line with government guidelines, **published in peer reviewed literature**  5: Publicly available system, developed in consultation with experts and in line with government guidelines (**not published in peer reviewed literature**)  2.5: Publicly available system with no details of development/alignment with government guidelines OR **not publicly available but developed in consultation with experts and aligned with government guidelines**  0: No information / poor alignment / does not have a system |
| M-FORM7.2 | S-FORM7.2 | If a proprietary product classification system has been developed, which products, nutrients and food characteristics are covered, and what are the details? | For information only |  |

| **Nutrition labelling and information (LABEL)** | | | | |
| --- | --- | --- | --- | --- |
| M-LABEL1 | S-LABEL1 | Does the company commit to providing on-pack information on added sugar content? | 2.5 | 2.5: Yes, on all relevant products  1: Yes, on some products  0: No |
| M-LABEL2 | - | Does the company provide **online nutrition information** for its products? *  **refers to own-brand products in the case of grocery retailers* | 10 | 10: Yes, comprehensive nutrition information (calories, sodium, saturated fat, total fat, sugar) for all products  7.5: Yes, comprehensive nutrition information (calories, sodium, saturated fat, total fat, sugar) for most (>80%) products  5: Comprehensive nutrition information for some (>50%) products  2.5: Limited nutrition information (i.e. does not include calories, sodium, saturated fat, total fat or sugar) for some (>50%) items  0: <50% of products or no information |
| - | S-LABEL2.1 | Does the company provide nutrition information (e.g., ingredients lists, nutrition facts tables) in a consistent manner for all products in **online stores**? | 20 | 20: Yes, comprehensive nutrition information (calories, sodium, saturated fat, total fat, sugar) for all products  15: Yes, comprehensive nutrition information (calories, sodium, saturated fat, total fat, sugar) for most (>80%) products  10: Comprehensive nutrition information for some (>50%) products  5: Limited nutrition information (i.e. does not include calories, sodium, saturated fat, total fat or sugar) for some (>50%) items  0: <50% of products or no information |
| - | S-LABEL2.2 | Does the company report on strategies to guide consumers to purchase healthier products in online settings? (e.g., displaying front of package labelling for products, implementing a product filter for ‘healthier’ products on its online store) | 10 | 10: Comprehensive set of strategies to guide consumers towards healthier purchases, with prominent features applied across the company website/online store. 5: Some strategies to guide consumers towards healthier purchases, with some features applied to some products online. 0: No strategies reported. |
| - | S-LABEL3.1 | Does the company commit to disclose **nutrition information** (e.g., on menus) for **takeaway or ready-to-eat foods** that are prepared on site? | 10 | Up to 10 points maximum: 5: Energy / calories 5: Symbol or logo indicating ‘healthy’ items 5: Sodium/salt 2: Saturated fat 2: Total fat 2: Trans fat 2: Sugar |
| - | S-LABEL3.2 | What information does the company commit to providing (e.g., on menus) for takeaway or ready-to-eat foods that are prepared on site? |  |  |
| - | S-LABEL3.3 | Is the nutrition information (e.g., on menus) for takeaway or ready-to-eat foods that are prepared on site presented in the same size and font as price? | 5 | 5: Yes 0: No |
| - | S-LABEL3.4 | If energy / calorie information is displayed on menus, does the company provide a contextual statement regarding the number of calories that should be consumed in a day for the average adult to maintain a healthy weight? | 5 | 5: Yes  0: No  n/a where mandated by government policy |
| - | S-LABEL4 | Does the company use **shelf tags** that provide summary nutrition information (e.g., Health Star Rating, traffic lights)? If so, which products is this applied to? Which stores use this system? | 10 | 10: Yes, labelling system used for all product categories, classification based on official national or regional classification system (developed by WHO, PAHO, national government, etc.) 7.5: Yes, labelling system used for some product categories, classification based on official national or regional classification system (developed by WHO, PAHO, national government, etc.) 5: Yes, labelling system used for all product categories, classification based on own system that has been validated and shows strong alignment with official national (or regional) classification systems / dietary guidelines, published in peer-reviewed literature 2.5: Yes, labelling system used for some product categories, classification based on own system that has been validated and shows strong / moderate alignment with official national (or regional) classification systems / dietary guidelines, not published in peer-reviewed literature 0 : No |
| - | S-LABEL5 | Does the company have an ongoing **nutrition/healthy eating education program in-store**? (e.g., dieticians in stores, nutrition education materials, etc.). To which stores does this apply? | 5 | 5: Yes, in all/most stores  2.5: Seasonal /intermittent programs only, or only selected stores  1: Actively considering/engaged in options for nutrition/healthy eating education programs  0: No information available to the research team |
| M-LABEL3.1 | S-LABEL6.1 | Does the company state that it will make a **nutrition content claim** (e.g., low in fat) on a product or as a part of advertising only when the product is 'healthy'? | 10 | 10: Yes, commitment is published  5: Yes, commitment is not published  0: No |
| M-LABEL3.2 | S-LABEL6.2 | What system / criteria (e.g., product classification system or nutrient profiling system) does the company use to classify the healthiness of products for the purposes of health and/or nutrition claims? | 10 | 10: Uses government guidelines/government endorsed classification system  7.5: Publicly available system, developed in consultation with experts and in line with government guidelines, published in peer reviewed literature  5: Publicly available system, developed in consultation with experts and in line with government guidelines (not published in peer reviewed literature)  2.5: Publicly available system with no details of development/alignment with government guidelines OR not publicly available but developed in consultation with experts and aligned with government guidelines  0: No information / poor alignment / does not have a system |

| **Product and brand promotion (PROMO)** | | | | |
| --- | --- | --- | --- | --- |
| M-PROMO1.1 | S-PROMO1.1 | Does the company have a policy to reduce the power and exposure of unhealthy food^2^ marketing to children on **broadcast media** (TV, radio)? | 10 | 10: Yes, national policy and noted on company website / annual reports  7.5: Yes, global policy and noted on company website / annual reports  5: Yes, national policy but not noted on company website / annual reports  2.5: Yes, global policy but not noted on company website / annual reports  0: No |
| M-PROMO1.2 | S-PROMO1.2 | To what age group does this broadcast media policy apply and how is the 'target audience' defined? (e.g., 12 years and under, when 35% or more of the audience is children) | 20 | 10: Under 18 years  8: 16 years and under  6: 14 years and under  4: 12 years and under  2: Under 10 years  0: No information  N/A: no policy   10: Time-based restrictions, based on children’s peak viewing times (e.g., no advertising before 9:00pm)  5: Based on audience share (e.g., if >10% of audience are children)  2.5: Children’s programs only  0: No explicit threshold / definition |
| M-PROMO2.1 | S-PROMO2.1 | Does the company have a policy to reduce the exposure of children to unhealthy food marketing on **digital/online media** (including websites and social media)? | 20 | 10: Yes, national policy and noted on company website / annual reports  7.5: Yes, global policy and noted on company website / annual reports  5: Yes, national policy but not noted on company website / annual reports OR national policy and noted on industry association website  2.5: Yes, global policy but not noted on company website / annual reports  0: No policy / no information available to the research team  Select all that apply (max 10 points): 5: Applies to digital content on company media (such as company website or social media) 5: Applies to all paid online marketing  N/A if no policy |
| M-PROMO2.2 | S-PROMO2.2 | To what age group does this digital/online marketing policy apply? | 10 | 10: Under 18 years  8: 16 years and under  6: 14 years and under  4: 12 years and under  2: Under 10 years  0: No policy / no information |
| M-PROMO3.1 | S-PROMO3.1 | Does the company have a policy to reduce the power and exposure of unhealthy food marketing to children on **other non-broadcast media** (including DVDs/games, print media, product placement, outdoor marketing, in store marketing, point of sales marketing)? | 10 | 10: Yes, national policy and noted on company website / annual reports  7.5: Yes, global policy and noted on company website / annual reports  5: Yes, national policy but not noted on company website / annual reports  2.5: Yes, global policy but not noted on company website / annual reports  0: No |
| M-PROMO3.2 | S-PROMO3.2 | To what age group does this non-broadcast media policy apply and how is the 'target audience' defined? (e.g., 12 years and under, when 35% or more of the audience is children) | 20 | 10: Under 18 years  8: 16 years and under  6: 14 years and under  4: 12 years and under  2: Under 10 years  0: No policy / no information  10: Time-based restrictions, based on children’s peak viewing times (e.g., no advertising before 9:00pm)  5: Based on audience share (e.g., if >10% of audience are children)  2.5: Children’s programs only  0: No explicit threshold / definition |
| M-PROMO4 | S-PROMO4 | Does the company have a commitment on the use of unhealthy brands in the **sponsorship** of children's sporting, cultural or other activities? | 10 | 10: Yes, including foods and brands OR no action in this area  5: Yes, foods only  0: No commitment |
| M-PROMO5 | S-PROMO5 | Does the company commit not to promote unhealthy brands (foods or company brands) in settings **where children gather**? | 10 | (Can be multiple)  2: Commits IN early childcare settings and primary schools (children up to age 11)  2: Commits NEAR (e.g. within 500m) of early childcare settings and primary schools (children up to age 11)  2: Commits IN secondary schools (children between age 12 and 18)  2: Commits NEAR (e.g., within 500m) of secondary schools (children between age 12 and 18)  2: Commits in other places where children gather (family and child clinics, paediatric services or other health facilities, sporting or recreation centres, or sporting or cultural events held at those premises) |
| M-PROMO6 | S-PROMO6 | Does the company have a commitment on the use of **celebrities** that appeal to children in the marketing of products? | 5 | 5: All forms of marketing  2.5: Some forms of marketing (e.g., excludes packaging) or applies only to those celebrities that appeal primarily to children  0: No policy / no information available to the research team |
| M-PROMO7 | S-PROMO7 | Does the company have a commitment on the use of **fantasy and animation characters** that appeal to children in the marketing of products? | 10 | 10: All forms of marketing (includes no use of characters with strong appeal to children across all forms of marketing)  5: Some forms of marketing (includes no use of characters with strong appeal to children across some forms of marketing)  2.5: Some restriction on use of fantasy/animation character in marketing (e.g. only applies to characters that appeal primarily to children or does not apply to licensed/third party characters)  0: No policy / no information available to the research team |
| M-PROMO8 | S-PROMO8 | Does the company have a commitment on the use of **premium offers** that appeal to children (e.g., promotional toys, games, vouchers and competitions) in the marketing of products? | 5 | 5: All forms of marketing  2.5: Some forms of marketing (e.g., excludes packaging)  0: No commitment / no information available to the research team |
| M-PROMO9.1 | S-PROMO9.1 | What system / criteria (e.g., product classification system or nutrient profiling system) does the company use to classify the healthiness of products for the purposes of promotion to children? | 10 | 10: Uses government guidelines/government endorsed classification system (where available)  7.5: Publicly available system, developed in consultation with experts and in line with government guidelines, published in peer reviewed literature  5: Publicly available system, developed in consultation with experts and in line with government guidelines (not published in peer reviewed literature)  2.5: Publicly available system with no details of development/alignment with government guidelines OR not publicly available but developed in consultation with experts and aligned with government guidelines  0: No information / poor alignment / does not have a system  **** 10/10 if no marketing regardless of nutritional profile.* |
| M-PROMO9.2 | S-PROMO9.2 | If a proprietary product classification system has been developed, which products, nutrients and food characteristics are covered, and what are the details? | For information only |  |
| M-PROMO10 | S-PROMO10 | Does the company audit its compliance with its policy on marketing to children at the national/country level? | 5 | 5: Yes, the policy is audited by an independently appointed third party  2.5: Internally audited but not independently audited or mandatory pre-clearance  1: Optional pre-clearance  0: No auditing is conducted  **DIVIDE POINTS BY HALF IF THE POLICY IS ONLY AUDITED AT THE GLOBAL LEVEL, AND NOT AT THE NATIONAL/COUNTRY LEVEL** |
| M-PROMO11.1 | S-PROMO11.1 | Does  the company have a **position in relation to government policies** for reducing the exposure of children and adolescents to the marketing of unhealthy foods?  Is this position statement published? | 10 | 10: Yes, on own website  5: Yes, on industry association website  0: Not publicly available |
| M-PROMO11.2 | S-PROMO11.2 | Does the company’s policy position support WHO’s position on government-led policy action related to reducing the exposure of children and adolescents to, and the power of, the marketing of unhealthy foods, as articulated in the WHO Global Action Plan for NCDs and other key WHO documents (such as the Report of the Commission on Ending Childhood Obesity)? | 10 | 10: Strong support (e.g., includes support for government-led action of marketing to children and adolescents, related to power and exposure)  5: Weak support (e.g., includes support for government-led action of marketing to children or adolescents, related to either power or exposure,)  0: No details available  -5: Somewhat opposed (e.g., opposes government-led efforts to restrict some aspects of promotion to children / adolescents)  -10: Strongly opposed (e.g., opposes any actions to reduce promotion to children) |
| M-PROMO12 | S-PROMO12.1 | Does the company have a responsible marketing policy or commitment that prioritizes healthy food and restricts marketing and advertising of unhealthy food to **all consumers**? | 10 | 10: Yes, national policy and noted on company website / annual reports   7.5: Yes, global policy and noted on company website / annual reports   5: Yes, national policy but not noted on company website / annual reports OR national policy and noted on industry association website   2.5: Yes, global policy but not noted on company website / annual reports OR vague public policy or commitment regarding the marketing of healthy foods to all  0: No policy/ no information available to the research team |
| - | S-PROMO12.2 | To which media / settings does the marketing policy (related to all consumers) apply (e.g., TV/Radio, websites, DVDs, games, print, social media)? | 15 | (Can be multiple) 5: Broadcast media (TV, Radio) 5: Online/digital (websites, social media) 5: Non-broadcast media (including, DVDs/games, print media, product placement, outdoor marketing) |
| M-PROMO13 | S-PROMO13 | Does the company report on the proportion of **marketing budget** spent on unhealthy and healthy products? | 10 | 10: Yes, published SMART target with regular reporting on proportion of marketing spend on healthy and unhealthy products at national level, by media channel and by age group  5: Yes, but target is not published, not SMART or only applies to some parts of their portfolio  2.5: General statement on marketing expenditures for healthier portfolio items  0: No information or no target |
| - | S-PROMO14 | Does the company have a policy to limit their **in-store promotion** of unhealthy products? | 10 | 10: Yes, published policy commits to only promote healthy products in-store 7.5: Yes, published policy commits to ensuring that a minimum proportion of in-store promotion is for healthy products 5: Policy exists, but not published 0: No policy / no information available to the research team |
| - | S-PROMO15 | Does the company have a policy on the proportion of healthy (compared with unhealthy foods) foods promoted in their regular **catalogues/circulars**? | 10 | 10: Yes, published policy commits to only promote healthy products in regular catalogues/circulars 7.5: Yes, published policy commits to ensuring that a minimum proportion of products promoted in regular catalogues/circulars is for healthy products 5: Policy exists, but not published 0: No policy / no information available to the research team |
| - | S-PROMO16 | Does the company have a policy to link **rewards programs or loyalty programs** to healthy food items? | 5 | 5: Yes, published policy commits to link rewards / loyalty schemes to healthy food products 2.5: Yes, published policy commits to link a proportion of rewards / loyalty schemes to healthy food products / not published 0: No policy / no information available to the research team |
| - | S-PROMO17 | Does the company have a policy to ensure that in-store product presentations, product giveaways or **tastings** are for healthy products? | 5 | 5: All presentations, product giveaways or tastings must be for healthy products 2.5: Some presentations or tastings (e.g. those aimed at children) must be for healthy products 0: No policy / no information available to the research team |
| - | S-PROMO18 | What **system / criteria** (e.g., product classification system or nutrient profiling system) does the company use to classify the healthiness of products for the purposes of product promotion to all consumers (e.g. in-store catalogues, brochures, flyers, shelf tags, promotional posters)? | 10 | 10: Uses government guidelines/government endorsed classification system (where available)  7.5: Publicly available system, developed in consultation with experts and in line with government guidelines, published in peer reviewed literature  5: Publicly available system, developed in consultation with experts and in line with government guidelines (not published in peer reviewed literature)  2.5: Publicly available system with no details of development/alignment with government guidelines OR not publicly available but developed in consultation with experts and aligned with government guidelines  0: No information / poor alignment / does not have a system |
| - | S-PROMO19 | Does the company commit to or provide evidence of incentivizing food manufacturers and suppliers to prioritize promotional activities on healthy foods/brands (as opposed to unhealthy foods/brands)?   For example, ensuring contract negotiations with manufacturers and suppliers include support for promotional activities of healthy foods/brands. | For information only | [Information only, not to be scored]  10: Yes, commitment or evidence of incentivizing manufacturers and suppliers as part of contractual requirements, across all categories. 5: Yes, commitment or evidence in some categories only  2.5: General commitment to encouraging manufacturers and suppliers  0: No evidence / policy / information |
| **Product accessibility (ACCESS)** | | | | |
| M-ACCESS1 | S-ACCESS1 | Does the company make a commitment to address the **price / affordability** of its healthier products relative to its unhealthy products? | 10 | 10: Commitment that standard prices of healthy products are lower than standard prices of comparable unhealthy products  7.5: Commitment that some healthier products will be priced lower than standard prices 5: Commitment that standard prices of healthy products are equivalent to standard prices of comparable unhealthy products  2.5: Broad commitment only  0: No commitment/ no information |
| - | S-ACCESS2 | Does the company have a published position on the **size and nature of discounts / price promotions** applied to healthy and unhealthy foods? | 10 | 10: Commitment to no price promotions on unhealthy foods, at any time of the year (including seasonal promotions, such as during major cultural festivals, where these strategies are often highly prevalent)  7.5: Commitment to greater levels of discount typically applying to healthy foods compared to unhealthy foods across all food categories 5: Commitment to the same types / levels of discounts typically applying on healthy and unhealthy foods across all food categories 2.5: Commitment to the same types / levels of discounts typically applying on healthy and unhealthy foods for some food categories 0: No policy / no information available to the research team |
| - | S-ACCESS3 | Does the company make a commitment to limit **multi-buy specials** (e.g., two for one) on unhealthy foods? | 10 | 10: Commitment to no multi-buy specials for unhealthy foods, at any time of the year (including seasonal promotions, such as during major cultural festivals, where these strategies are often highly prevalent)  5: Commitment to limit multi-buy specials for unhealthy foods 0: No commitment / no information available to the research team |
| M-ACCESS2.1 | S-ACCESS4.1 | Does the company have a **position in relation to government fiscal policies** with respect to making healthier foods relatively cheaper and unhealthy foods relatively more expensive? | 10 | 10: Yes, on own website  5: Yes, on industry association website  0: Not publicly available  * Both indicators were scored together (total possible points = 10). |
| M-ACCESS2.2 | S-ACCESS4.2 | Is this position statement published? |  |  |
| M-ACCESS2.3 | S-ACCESS4.3 | Does the company’s policy position support WHO’s position on fiscal policies to make healthier foods relatively cheaper and unhealthy foods relatively more expensive, as articulated in the WHO Global Action Plan for NCDs and the Report of the Commission on Ending Childhood Obesity, Recommendation 1.2)? | 10 | 10: Strong support (e.g., includes support for taxes on unhealthy foods or sugar sweetened beverages, broadly defined, as well as subsidies for healthy foods)  5: Weak support (e.g., includes support for taxes on unhealthy foods or sugar-sweetened beverages, narrowly defined, or subsidies for healthy foods)  0: No details available  -5: Somewhat opposed (e.g., opposes taxes on unhealthy foods or subsidies for healthy foods)  -10: Strongly opposed (e.g., opposes all measures in this area) |
| M-ACCESS3 | S-ACCESS5 | Does the company make a clear and specific commitment to increase the availability of healthy products, or to decrease the **availability** of unhealthy products in specific settings? | 10 | 10: Yes, publicly available commitment to increase availability of healthier products for whole of business or over a range of key settings (e.g., including remote communities, schools, hospitals and community events)  7.5: Yes, internal commitment for whole of business over a range of key settings that is not published  5: Public commitment for some settings (e.g, schools, remote communities, community events or hospitals)  2.5: Commitment for some settings (e.g, schools, remote communities, community events or hospitals) that is not published, or broad commitment only  0: No commitment / no information available to the research team |
| - | S-ACCESS6 | Does the company make a clear and specific commitment to dedicate a minimum amount or proportion of **shelf space or floor space** to healthy products? | 10 | 10: Clear commitment for whole business, and is published  7.5: Clear commitment for whole business, is not published  5: Broad commitment, is published  2.5: Broad commitment, is not published  0: No commitment / no information available to the research team |
| - | S-ACCESS7 | Does the company have a policy that **checkouts** are free from unhealthy items (including confectionery, chocolate and soft drinks)? | 10 | 10: No unhealthy items, applies to all checkouts  7.5: No unhealthy items, applies to some checkouts OR limit unhealthy items, applies to all checkouts  5: Limit unhealthy items, applies to some checkouts  2.5: Actively considering/engaged in healthy checkout options  0: No policy / no information available to the research team |
| - | S-ACCESS8 | Does the company have a published **position on the placement of unhealthy items** (such as confectionery, chocolate and soft drinks) at end of aisle displays or other high-traffic areas? | 10 | 10: No unhealthy items, applies to all high-traffic areas  7.5: No unhealthy items, applies to some high-traffic areas  5: Limit unhealthy items, applies to all high-traffic areas  2.5: Limit unhealthy items, applies to some high-traffic areas  0: No commitment / no information available to the research team |
| M-ACCESS4.1 | S-ACCESS9.1 | What **system / criteria** (e.g., product classification system or nutrient profiling system) does the company use to classify the healthiness of products for the purposes of food pricing, distribution and/or availability? | 10 | 10: Uses government guidelines/government endorsed classification system (where available)  7.5: Publicly available system, developed in consultation with experts and in line with government guidelines, published in peer reviewed literature  5: Publicly available system, developed in consultation with experts and in line with government guidelines (not published in peer reviewed literature)  2.5: Publicly available system with no details of development/alignment with government guidelines OR not publicly available but developed in consultation with experts and aligned with government guidelines  0: No information / poor alignment / does not have a system |
| M-ACCESS4.2 | S-ACCESS9.2 | If a proprietary product classification system has been developed, which products, nutrients and food characteristics are covered, and what are the details? | For information only |  |
| - | S-ACCESS10 | Does the company commit to or provide evidence of working with food manufacturers and suppliers to ensure their healthy products are affordable, accessible, and distributed equitably?  For example, ensuring contract negotiations with manufacturers and suppliers support sales of their healthy or healthier products, including through shelf space provision, prominent placement and wide distribution. | For information only | [Information only, not to be scored]  10: Yes, commitment or evidence of incentivising manufacturers and suppliers as part of contractual requirements, across all categories. 5: Yes, commitment or evidence in some categories only  2.5: General commitment to encouraging manufacturers and suppliers  0: No evidence / policy / information |

| **Disclosure of relationships with external organizations and lobbying (RELAT)** | | | | |
| --- | --- | --- | --- | --- |
| M-RELAT1 | S-RELAT1 | Does the company publish details of the professional organisations (e.g., professional associations for nutrition or dietetics, physical activity or exercise organisations, medical organisations or societies, etc.) and/or scientific events (e.g., conferences) it funds or supports, including awards/prizes, making clear the nature of that support? Please provide details of which professional organisations are supported by the company. | 10 | 10: Yes, information on national-level activity is publicly available (website or document) in a consolidated and cumulative form  5: Yes, information is available, but is not consolidated and easy to locate OR information is available at the global level only OR comprehensive information about their activities in the area provided to the project team  0: No information available / provided  N/A: No activity in this area (subtract 10 from overall possible score for this section) |
| M-RELAT2 | S-RELAT2 | Does the company publish details of the external research (e.g., conducted by individuals/groups/organisations) it funds or supports, including awards/prizes? Please provide details of which external research groups receive support from the company. | 10 | 10: Yes, information on national-level activity is publicly available (website or document) in a consolidated and cumulative form  5: Yes, information is available, but is not consolidated and easy to locate OR information is available at the global level only OR comprehensive information about their activities in the area provided to the project team  0: No information available / provided  N/A: No activity in this area (subtract 10 from overall possible score for this section) |
| M-RELAT3 | S-RELAT3 | For philanthropic funding, does the company publish details of the groups or organisations it funds or supports? Please provide details of which philanthropic groups receive support from the company. | 10 | 10: Yes, information on national-level activity is publicly available (website or document) in a consolidated and cumulative form  5: Yes, information is available, but is not consolidated and easy to locate OR information is available at the global level only OR comprehensive information about their activities in the area provided to the project team  0: No information available / provided  N/A: No activity in this area (subtract 10 from overall possible score for this section) |
| M-RELAT4.1 | S-RELAT4.1 | Does the company publish details of the nutrition education / healthy diet oriented programs it funds or supports? Please provide details of which nutrition education programs receive support from the company. | 10 | 10: Yes, information on national-level activity is publicly available (website or document) in a consolidated and cumulative form  5: Yes, information is available, but is not consolidated and easy to locate OR information is available at the global level only OR comprehensive information about their activities in the area provided to the project team  0: No information available / provided  N/A: No activity in this area (subtract 10 from overall possible score for this section) |
| M-RELAT4.2 | S-RELAT4.2 | For nutrition education / healthy diet oriented programs, does the company have a commitment to align programs to national or regional dietary guidelines? | For information only |  |
| M-RELAT5 | S-RELAT5 | Does the company publish details of the active lifestyle programs (sports, physical activity) it funds or supports? Please provide details of which active lifestyle programs receive support from the company. | 10 | 10: Yes, information on national-level activity is publicly available (website or document) in a consolidated and cumulative form  5: Yes, information is available, but is not consolidated and easy to locate OR information is available at the global level only OR comprehensive information about their activities in the area provided to the project team  0: No information available / provided  N/A: No activity in this area (subtract 10 from overall possible score for this section) |
| M-RELAT6 | S-RELAT6 | Does the company publish details of its involvement in public-private partnerships and/or joint ventures with government organisations / agencies? Please provide details of which public-private partnerships the company is involved in. | 10 | 10: Yes, information on national-level activity is publicly available (website or document) in a consolidated and cumulative form  5: Yes, information is available, but is not consolidated and easy to locate OR information is available at the global level only OR comprehensive information about their activities in the area provided to the project team 0: No information available / provided N/A: No activity in this area (subtract 10 from overall possible score for this section) |
| M-RELAT7 | S-RELAT7 | Does the company publish details of its political donations? | 10 | 10: Yes, information on national-level activity is publicly available (on a company website or document) OR declaration of no activity in this area 0: No |
| M-RELAT8 | S-RELAT8 | Does the company publish its membership/ support for/ ownership of industry associations, think tanks, interest groups, community organisations or other organisations that lobby in relation to population nutrition and/or obesity and NCDs issues? Please provide details of which industry associations the company is involved in. | 10 | 10: Yes, information on national-level activity is publicly available (website or document) in a consolidated and cumulative form  5: Yes, information is available, but is not consolidated and easy to locate OR information is available at the global level only OR comprehensive information about their activities in the area provided to the project team 0: No information available / provided N/A: No activity in this area (subtract 10 from overall possible score for this section) |
| M-RELAT9 | S-RELAT9 | Does the company make publicly available its submissions (or submissions with which the company is associated, such as through industry associations) to public consultations regarding relevant population nutrition policies? | 10 | 10: Yes (on company websites or in a document that is publicly available upon request) 0: No |

^1^ SMART targets and significant past action for product reformulation were considered if targets were set for and reported on within the past 5 years (i.e., since 2018). FORM indicators were deemed not applicable on a case-by-case basis if a company’s portfolio did not contain products for which the nutrient might be considered of public health concern (e.g., a beverage only company that only produces sugary beverages would not be assessed for having sodium-related reformulation targets).^2^ Throughout this table, the term ‘food’ encompasses both foods and beverages.
